# Cytokine Expression in Canine Lymphoma, Osteosarcoma, Mammary Gland Tumour and Melanoma: Comparative Aspects

**DOI:** 10.3390/vetsci6020037

**Published:** 2019-04-02

**Authors:** Sergio Erdal Irac, Annika Oksa, Karen Jackson, Aaron Herndon, Rachel Allavena, Chiara Palmieri

**Affiliations:** School of Veterinary Science, The University of Queensland, Gatton, QLD 4343, Australia; s.irac@uq.edu.au (S.E.I.); a.oksawalker@uq.edu.au (A.O.); karen.jackson@uq.edu.au (K.J.); a.herndon@uq.edu.au (A.H.); r.allavena@uq.edu.au (R.A.)

**Keywords:** osteosarcoma, melanoma, lymphoma, mammary gland tumours, cytokines

## Abstract

Cytokines released in the tumour microenvironment play a major role in cancer pathogenesis. In human cancers and corresponding animal models, cytokine expression contributes to tumour growth and progression, as well as regulation of the host anti-tumour response. The elucidation of the function and importance of cytokines in canine cancers is still in an early stage, although relevant data have been obtained in classical examples of comparative models of human cancers, such as osteosarcoma, melanoma, mammary tumour and lymphoma. A deeper understanding of the cytokine signature may advance diagnosis, prevention and treatment of canine cancers.

## 1. Introduction

Cancer is a devastating disease affecting both humans and their most beloved companions, dogs. It is the leading cause of death in people in developed countries, and the second leading cause of death in developing nations. Cancer is also the leading cause of death in pet dogs, with an estimated four million dogs affected worldwide per year [1]. The study of carcinogenesis and the development of different cancer therapies is an extremely rapidly advancing area of research. Gold standard treatments have traditionally involved surgery, chemotherapy, and radiotherapy. We now understand the role of the immune system in carcinogenesis in much more detail, and an expanding number of human cancer treatment protocols incorporate immunotherapy. Dogs act as an excellent translational model for studying cancer and the tumour microenvironment, as they develop spontaneous cancers that demonstrate similar clinical behaviour to their human counterparts, recapitulate the biology and heterogeneity of the human disease, including interactions between the immune system and tumour cells, and their response to treatment is comparable [2,3,4]. This review will investigate the current knowledge of cytokines in canine cancer pathogenesis from a comparative aspect, focusing on those cancers that represent excellent translational models: lymphoma, melanoma, mammary gland tumour, and osteosarcoma.

## 2. Role of Cytokines in Cancer Development 

Cytokines are small proteins that act as cell-to-cell messengers and play an important role within the immune system by stimulating or inhibiting cells in response to a range of stresses. They also control the differentiation, activation and growth of different cell types [5]. Cytokines are therefore critical in host defence as well as normal physiological and metabolic processes. Thus, they integrate de facto into the host defence and metabolic functions [5]. The effects of individual cytokines on immunity depend on the local cytokine concentration, the expression of specific receptors and the activation of multiple signalling pathways. Cytokine signalling is characterised by a significant degree of pleiotropism, in which one cytokine can act on many different cell types to mediate diverse and sometimes opposing effects. They are often produced in a cascade, as one cytokine stimulates its targeted cell to secrete additional cytokines. They may act synergistically or antagonistically [5]. Most of the information regarding the role of cytokines in the tumour microenvironment derives from murine and human studies. Cytokines can be produced by both neoplastic cells and the tumour microenvironment, including reactive lymphocytes, dendritic cells (DC) and macrophages, and they play an important role as mediators and/or regulators of tumour–host interactions leading to the progression of malignancies. During carcinogenesis, they are involved in apoptosis, angiogenesis, cell adhesion and transformation [6,7]. The interaction between a tumour and the immune system and the production of cytokines by a tumour itself can result in high systemic levels of cytokines in cancer patients [7,8] (Table 1), suggesting that peripheral blood cytokines may be potentially used as diagnostic biomarkers.

In addition, variations in the gene sequence (polymorphism) of different cytokines have been associated with increased risk of specific human cancers [25,26,27,28,29]. Identification of individuals at risk allows for early detection, proper treatment and management of the disease, potentially reducing incidence, morbidity, and mortality. Genetic polymorphism may also play a significant part in determining the response of patients to anti-cancer immunotherapy. 

Even without a comprehensive theory of the entire immune reaction pattern, several cytokine alterations have been addressed explicitly in various experimental treatments and clinical trials. TNF, IFN and IL-2 have been used to treat a variety of a human solid tumours, including melanoma, renal cell carcinoma, multiple myeloma and ovarian cancer [30]. Moreover, the ability of cytokine-secreting tumours to function as a cellular vaccine is under investigation [31]. 

## 3. Cytokines and Lymphoma

Lymphoproliferative disorders (LPDs) are common in dogs, with canine non-Hodgkin lymphoma (NHL) having the highest incidence [32]. The biological behaviour of NHL is similar between dogs and people, including genomic instability, response to treatment and the possible involvement of heritable risk factors [2,33]. Despite being one of the most common cancers in dogs, our understanding of the pathogenesis and in particular how neoplastic cells shape the microenvironment for their survival and progression is still limited. 

In humans, some cytokines are considered useful diagnostic biomarkers for NHL, since they are significantly increased in the serum of cancer-affected patients compared to controls: including vascular endothelial growth factor (VEGF), interleukin-6 (IL-6), IL-8, IL-10, IL-13, chemokine ligand 9 (CXCL9), interferon gamma-induced protein 10 (IP-10), interferon-gamma (IFN-γ), IL-12p40 (the p40 subunit of IL-12), macrophage inflammatory protein-1α/β (MIP-1α/β), and hepatocyte growth factor (HGF) [12,34,35]. 

Moreover, a few cytokines possess biologic predictive and therapeutic significance. High serum levels of IL-6 has been associated with a shorter overall survival (OS) and a lower complete response (CS) rate in human lymphoma [13,14,36]. In Hodgkin’s disease, IL-6 has been shown to return to normal in patients who achieve a complete response after treatment [35]. In the first week after CHOP (Cyclophosphamide, Doxorubinc, Vincristine, Prednisone) therapy, if the treatment is successful, serum VEGF levels will significantly decrease [36].

Canine lymphoma represents the most well-studied cancer in terms of cytokine or blood biomarker involvement. Dogs with lymphoma have higher circulating macrophage chemoattractant protein-1 (MCP-1), VEGF, IL-6, IL-10 and lower transforming growth factor-β (TGF- β) than healthy dogs [15,16,17,18,37]. VEGF is higher in T cell lymphomas than B cell lymphomas, and in high grade lymphoma compared to intermediate or low grade lymphoma [15]. B and T cell lymphomas have a different serum cytokine signature, with T cell lymphoma associated with increased levels of IL-6, whilst IL-10 is more prominent in B cell lymphoma–affected canine patients [37]. Serum tumor necrosis factor-α (TNF-α) appears to have a limited value as a tumour marker in dogs with lymphoma, being detectable in only 12% of affected patients [38]. After chemotherapy, VEGF decreases in canine B-cell lymphoma [15], while elevated baseline serum MCP-1 is associated with a lower disease-free interval in dogs treated with the CHOP (cyclophosphamide, vincristine, doxorubicin, prednisone) protocol [18]. Pretreatment MCP-1 concentrations are also significantly correlated with disease stage, with dogs presenting with stage III having a lower serum MCP-1 level compared to stage IV-V [18]. 

## 4. Cytokines and Osteosarcoma

Osteosarcoma (OSA) is the most common bone cancer in dogs. The biological behaviour of canine OSA is very aggressive and despite therapeutic advancements, survival times have not changed in the past 20 years. More than 80% of OSA-affected patients are diagnosed with lung metastasis [39] and with current standard of care consisting of limb amputation or limb-sparing surgery and chemotherapy the median survival time is 8–12 months [40]. Even in humans, the most commonly diagnosed primary bone tumour is osteosarcoma (over 56% of all bone tumours), representing the third most frequent cancer in adolescents. Although the canine disease is much more aggressive than the human, the prognosis for human patients with metastatic OSA is also poor with only 20% surviving event-free for five years post-diagnosis [41] and more than 30% not responding to chemotherapy [42]. Dogs are considered valuable models for human OSA with similar histology, response to treatment, distribution, genetic features and DNA structural changes. 

The autocrine and paracrine effects of several cytokines and growth factors have been extensively demonstrated in human OSA, as well as their involvement in OSA pathogenesis and progression, and in orchestrating the immune response to this cancer [11]. In addition to classic OSA cytokines (IL-6, VEGF, TGF-β, interferons, TNF-α, IL-2), new players have been identified in the emerging cytokine network (IL-15, IL-17, IL34, IL-8, CXCL12, fractalkine) providing meaningful information relevant to osteosarcoma immunotherapy [11]. 

Several studies have highlighted an important role of TGF-β in the aggressive behaviour of human and canine OSA. All the isoforms of TGF-β (TGF-β 1–3) are implicated in bone formation, remodelling and bone tumour metastasis [43]. TGF-β1 tumour expression is directly correlated to human OSA grade, while levels of TGF-β3 are inversely related to disease-free survival [24]. Active secretion of TGF-β1 was demonstrated in three canine OSA cell lines [24]. Blocking the TGF-β signalling through a selective TGFβRI/II inhibitor (LY2109761) in canine OSA cell lines results in reduced cell proliferation, migration and VEGF secretion [24]. Therefore, in dogs TGF-β signalling might contribute to OSA progression and hence blockade of TGF-β mediated effects could be a rational therapeutic strategy. 

Using cross-species genomic analysis, Paoloni et al. (2009) [19] have demonstrated that the similarities between the genetic signatures of canine and paediatric osteosarcoma is strong and cluster analysis of selected transcripts could not distinguish the cancers based on species alone. In particular, two “dog-like” genes consistently expressed in canine OSA (IL-8 and SLC1A3) are linked to a more aggressive clinical course and poorer outcome in human osteosarcoma.

## 5. Cytokine and Mammary Gland Tumours

Canine mammary tumours (CMT) are the most common cancer among female dogs [44,45], representing approximately 53% of total neoplams. Many similarities have been identified between human breast cancer and certain canine mammary tumours, such as hormonal aetiology, histopathologic features of specific subtypes of carcinomas (e.g., canine simple carcinoma), molecular pattern, course of the disease, clinical stages and occurrence of premalignant lesions [3]. However, although there is a great potential as a canine model of human breast cancer, canine mammary cancer is poorly characterised at the genome-wide level and a large number of molecular pathways and genes mediating the development and progression of this tumour have not been well studied in such model. Therefore, further investigations are required to confirm if canine mammary tumours may faithfully recapitulate molecular features of human breast cancer.

In dogs with mammary carcinoma, serum IL-8 levels may be significantly increased compared to healthy animals, and positively correlated with tumour progression, lymph node involvement, recurrence and death [46]. This parallels similar findings observed in breast cancer patients where high IL-8 serum levels are considered an important poor prognostic factor [20]. IL-8 triggers breast cancer progression through its mitogenic and angiogenic properties [20]. IL-8 is also correlated with the metastatic phenotypes of human breast cancer cells [47] and stimulates bone metastasis [48]. The expression of IL-1, IL-6, TNF-α, and IFN-α are also higher in malignant and metastatic canine mammary carcinomas compared to benign mammary tumours [9,10]. De Andres et al. (2013) [21] have demonstrated increased serum levels and tissue expression of IL-8 and IL-10 in dogs with inflammatory mammary carcinoma compared with the non-inflammatory malignant mammary tumours. Canine inflammatory mammary cancer is characterised by a sudden onset and an aggressive clinical course, and despite being quite rare in dogs, has been proposed as the best animal model for studying human inflammatory breast cancer [49,50]. In human breast cancer patients, IL-8 and IL-10 expression levels are abnormally high and this has been associated with advanced clinical stage and poor prognosis [22,47]. 

The role of TGF-β in human breast cancer is controversial, being a potent inhibitor of cellular proliferation in the early stages of carcinogenesis and pro-oncogenic in advanced neoplastic progression through permissive effects on stromal tissue, angiogenesis and the immune system [51,52]. Canine mammary carcinomas and their metastases have significantly decreased expression levels of TGF-β and latent TGF-β binding protein 4 (LTBP4) than non-neoplastic mammary glands and canine mammary adenomas [23], suggesting that loss of their expression may have growth-stimulatory effects in advanced tumours. 

## 6. Cytokines and Melanoma

Melanoma is a relatively common tumour in dogs, occurring most often in the oral cavity and less frequently in the skin, eye, foot pads, nail bed (acral) and other mucocutaneous sites [53,54]. Most oral/mucosal and acral melanomas have a poor prognosis with a median survival time from three to 18 months [54]; however, in contrast canine cutaneous melanoma is often benign and cured with complete surgical excision. 

Canine and human malignant melanoma share common histological and clinical features, with similar propensity to metastasise to lymph nodes, brain and other visceral organs [54], as well as similar resistance to chemotherapy and radiation therapy [4]. 

Diagnostic potential and prognostic significance of cytokines in human melanoma have already been documented [55] with the first cancer patient responding to the administration of IL-2 in 1984 and remaining disease-free for the past 29 years [56]. Since then, IL-2 has been considered the first effective immunotherapy for human cancer.

Despite the lack of data on tissue expression and serum cytokine levels in canine melanoma, cytokine-based immunotherapy for canine melanoma represents a major growth area, with few trials completed or underway [57,58].

In particular, IL-2 in combination with granulocyte-macrophage colony-stimulating factor (GM-CSF) secreted by irradiated transgenic xenogeneic cells has been successfully used in dogs with malignant oral melanoma, with 80% of tumour mass loss and a higher percentage of metastasis-free patients [59]. 

In order to increase circulating half-life and decrease immunogenicity [60], an alternative cytokine treatment with TNF-α administered in a polyethylene glycol (PEG) was trialled in dogs with different tumours, including melanoma, although only a minor/transient antitumor response was demonstrated. 

## 7. Conclusions

**Figure 1:**
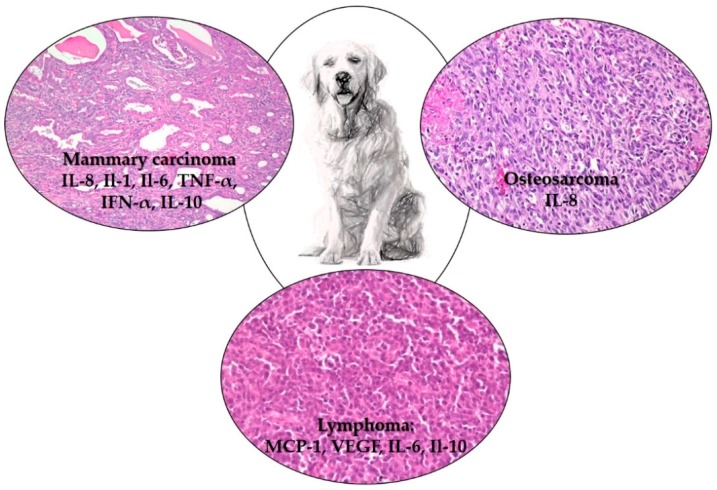
Cytokines with a potential diagnostic application in canine mammary carcinoma, osteosarcoma and lymphoma. Cytokines have been selected based on the literature [10,15,16,17,18,19,21,46]. All the cytokines are increased in the tumours included in the figure.

Over the past few years, evidence has accumulated that cytokines are dynamically involved in multiple aspects of canine cancers, although further investigations are required to clearly define their pro- or anti-tumourigenic potential. However, the information provided thus far is paving the way for new diagnostic, prognostics/predictive and therapeutic strategies (Figure 1). 

Adding serum levels of selected cytokines to diagnostic options in canine cancer patients would allow a better prognostic evaluation and would assist therapeutic decisions. Moreover, cytokines may give reliable information on the efficacy of therapy and very early response. As knowledge on the biological roles of cytokines in canine cancers increases, improved prognostication and therapy is expected to improve the outcomes of canine patients with cancer.

## Figures and Tables

**Table 1:** Comparative cytokine expression in serum and tissue samples of human and canine cancers.

Cytokine	Expression Level	Cancer Type
**IL-1**	Increased	Mammary carcinoma (D) [9,10]
**IL-2**	Increased	OSA (H) [11]
**IL-6**	Increased	Lymphoma (H, D) [12,13,14,15,16,17,18], OSA (H) [11], Mammary Carcinoma (D) [9,10]
**IL-8**	Increased	Lymphoma (H) [12,13,14], OSA (H, D) [11,19], Mammary carcinoma (H, D) [20,21]
**IL-10**	Increased	Lymphoma (H, D) [12,13,14,15,16,17,18], Mammary carcinoma (H, D) [9,21,22]
**IL-13**	Increased	Lymphoma (H) [12,13,14]
**VEGF**	Increased	Lymphoma (H, D) [12,13,14,15,16,17,18], OSA (H) [11]
**CXCL9**	Increased	Lymphoma (H) [12,13,14]
**IP-10**	Increased	Lymphoma (H) [12,13,14]
**IFN-γ**	Increased	Lymphoma (H) [12,13,14]
**IFN-α**	Increased	Mammary carcinoma (D) [9]
**IL-12p40**	Increased	Lymphoma (H) [12,13,14]
**MIP-1α/β**	Increased	Lymphoma (H) [12,13,14]
**HGF**	Increased	Lymphoma (H) [12,13,14]
**MCP-1**	Increased	Lymphoma (D) [15,16,17,18]
**TGF-β**	Decreased	Lymphoma (D) [15,16,17,18], Mammary carcinoma (D) [23]
	Increased	OSA (H) [11], OSA cell lines (D) [24]
**TNF-α**	Increased	OSA(H) [11], Mammary carcinoma (D) [9]

Legend: H = human; D = dog; OSA = osteosarcoma.

## Abbreviations

(CMT)	Canine mammary tumours
(CXCL9)	Chemokine Ligand 9
CX3CL1	chemokine (C-X3-C motif) ligand 1
FK	fractalkine
(HGF)	Hepatocyte Growth Factor
IFN	interferon
IFN-α/β	interferon alpha/beta
IFN-λ	interferon gamma receptor
IL-1	interleukin-1
IL-2	interleukin-2
IL-6	interleukin-6
IL-8	interleukin-8
IL-10	interleukin-10
IL-12	interleukin-12
IL-13	interleukin-13
IL-15	interleukin-15
IL-17	interleukin-17
IL-34	interleukin-34
(IP-10)	Interferon gamma-induced protein 10
IL-12p40	(the p40 subunit of IL-12)
(LPDs)	Lymphoproliferative disorders
(Mip-1α/β)	Macrophage inflammatory protein-1α/β
M-CSF	macrophage-colony stimulating factor
CSF-1	colony stimulating factor
(MCP-1)	Macrophage Chemoattractant Protein-1
(NHL)	non-Hodgkin lymphoma
OSA	osteosarcoma
PEG	polyethylene glycol
TGFβ	transforming growth factor beta
(TNF-α/β)	Tumor Necrosis Factor-α/β
(LTBP4)	TGFβ and latent TGFβ binding protein 4
(LY2109761)	TGFβRI/II inhibitor
(VEGF)	Vascular endothelial growth factor
